# Chronic Periprosthetic Hip Joint Infection. A Retrospective, Observational Study on the Treatment Strategy and Prognosis in 130 Non-Selected Patients

**DOI:** 10.1371/journal.pone.0163457

**Published:** 2016-09-22

**Authors:** Jeppe Lange, Anders Troelsen, Kjeld Søballe

**Affiliations:** 1 Lundbeckfoundation Centre for Fast-track Hip and Knee Surgery, Aarhus, Denmark; 2 Orthopaedic Research Unit, Aarhus University Hospital, Aarhus, Denmark; 3 Interdisciplinary Research Unit, Center for planned Surgery, Silkeborg Regional Hospital, Silkeborg, Denmark; 4 Department of Orthopaedic Surgery, Copenhagen University Hospital Hvidovre, Hvidovre, Denmark; Harvard Medical School/BIDMC, UNITED STATES

## Abstract

**Introduction:**

Limited information is available regarding the treatment strategy and prognosis of non-selected patients treated for chronic periprosthetic hip joint infection. Such information is important as no head-to-head studies on treatment strategies are available. The purpose of this study is to report on the treatment strategy and prognosis of a non-selected, consecutive patient population

**Methods:**

We identified 130 patients in the National Patient Registry, consecutively treated for a chronic periprosthetic hip joint infection between 2003–2008 at 11 departments of orthopaedic surgery. We extracted information regarding patient demographics, treatment and outcome. 82 patients were re-implanted in a two-stage revision (national standard), the remaining 48 were not re-implanted in a two-stage revision. We were able to collect up-to-date information on all patients to date of death or medical chart review with a minimum of 5 years follow-up by the nationwide electronic patient record system

**Results:**

After primary revision surgery, 53 patients (41%) had a spacer in situ, 64 (50%) had a resection arthroplasty and 13 (9%) did not have the infected implant removed. 63% were re-implanted in a two-stage revision. Re-implantation was performed after an interim period of 14 weeks (IQR 10–18). Patients re-implanted were younger (p-value 0.0006), had a lower CCS score (p-value 0.005), a lower ASA score (p-value 0.0001) and a 68% lower mortality risk in the follow-up period (p-value <0.00001). After adjusting for selected confounders, the mortality risk was no longer significantly different. The 5-year re-infection rate after re-implantation was 14.6% (95%CI 8.0–23.1). Re-infections occurred mainly within 3 years of follow-up. The overall 1-year survival rate was 92% (95%CI 86–96) and the overall 5-year survival rate was 68% (95%CI 59–75). The 5-year survival rate after a two-stage revision was 82% (95%CI 71–89) and in those not re-implanted 45% (95%CI 30–58).

**Conclusion:**

We found that patients who receive a two-stage revision after a chronic periprosthetic hip joint infection are younger and healthier when compared to those who do not receive a two-stage revision in a non-selected patient population, indicating a clear selection of patients into this treatment strategy. Re-infection rates following two-stage revision were comparable to international results. We found a high mortality rate in our study population, but the causality of death and chronic periprosthetic hip joint infection cannot be established in this study and this needs further attention.

## Introduction

Periprosthetic hip joint infection (hip PJI) continues to be a severe complication with a 5-year incidence rate exceeding one percent following primary procedures[1]. In the United States of America nearly 8000 registered revision for hip PJI were performed in 2006[2].

Globally, the most performed treatment of chronic hip PJI is a delayed reimplantation procedure, a two-stage revision, with clinical success rates reported as high as 90%[3–5]. Yet, current literature has not proven any re-implantation strategy clinically superior, and no head-to-head studies have been conducted[3].

As such, studies on the prognosis following treatment for chronic hip PJI report on highly selected cohorts following specific, non-controlled treatment procedures. Only limited information is available on larger, non-selected samples of patients with chronic hip PJI[6].

The availability of such information is important in assessing results from observational studies, especially when these are used to compare treatment strategies[3]. Knowledge of patients in non-selected cohorts yields important information to clinicians who aim to decide the most optimal treatment strategy in an everyday clinical setting.

The purpose of this study was to evaluate the treatment strategy and describe the prognosis of a non-selected sample of patients treated for chronic hip PJI.

## Patients and Methods

This study was performed as a multi-center, retrospective observational study utilizing an historical cohort of patients undergoing inpatient treatment for chronic hip PJI.

Patients were identified if registered in the Danish National Patient Registry from 2003 to 2008 at 11 departments of orthopaedic surgery as part of a previously reported data extraction[7]. The participating departments performed approximately 33% of all primary hip joint replacements (7998 performed nationwide) and 37% of all revisions (1304 performed nationwide) in 2008.

A relevant case-mix distribution based on gender, age, hip disease, Charnley category and co-morbidity[8] in these centres was believed to ensure internal and external validity.

Access to joint replacement surgery was subject to free and universal healthcare coverage for all patients in the study. The individual treatment strategy was performed at the discretion of the treating orthopaedic surgeon in collaboration with the patient. All performing orthopaedic surgeons were specialized in adult lower limb reconstructive surgery. No differences in treatment strategy or re-infection rates were identified between the participating hospitals.

A total of 461 patients were identified with a World Health Organizations International Classification of Disease 10^th^ revision (ICD-10) discharge diagnosis code T84.5 (*Infection and inflammatory reaction due to internal joint prosthesis*) in combination with any hip-joint specific Nordic Medico-Statistical Committee classification of surgical procedures code or with a hip-joint infection-specific code independently of ICD-10 code. Description of the codes has previously been published[7].

**Fig 1:**
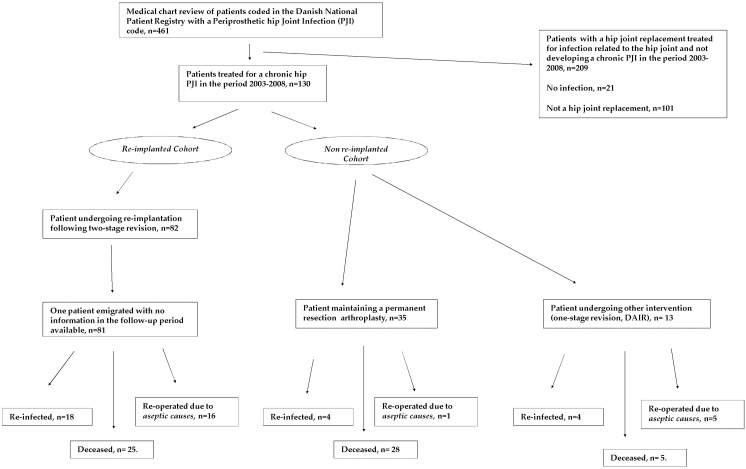
Patient flowchart. Flowchart of the 130 patients included in the study.

Among the 461 identified patients, 130 were verified as treated for a chronic hip PJI (see Fig 1).

A diagnosis of chronic hip PJI was adapted by the authors from the definition published by the workgroup of the American Musculoskeletal Infection Society[9], and defined as chronic by symptom duration of more than four weeks[10]. The definition used in this study is shown in Table 1[11].

**Table 1:** Definition of periprosthetic hip joint infections in the 130 identified patients.

• Category A:	Fistula to the prosthesis
• Category B:	Growth of identical microorganism in 3 to 5 of 5 separately taken per-operative tissue biopsies (the Kamme-Lindberg principle^16^)
• Category C:	3 or more of the following criteria: Growth of microorganism in cultures from joint fluid aspiration Growth of microorganism in per-operative tissue biopsies not defined as category B Visual pus or purulent fluid during exchange procedure (surgeon’s description) Radionuclide imaging procedure indicating infection Elevated C-Reactive Protein AND/OR Erythrocyte Sedimentation Rate Conventional X-ray of the hip indicating infection

The 130 patients were divided into two groups based on the performed treatment (see Fig 1): one group in which patients underwent re-implantation in a *two-stage* revision procedure, the national standard of practice at the time of the study (*re-implanted cohort*, n = 82); and a second group in which patients did not undergo a two-stage revision procedure (*Non re-implanted cohort*, *n = 48)*. This group consisted of 35 patients with permanent resection arthroplasty, one patient kept on suppressive antibiotics without surgical intervention, one patient with a direct exchange of implants and 11 patients with only debridement performed.

We excluded hip joint replacements with ongoing treatment for a chronic infection initiated prior to the inclusion period and not concluded at the initiation of the inclusion period, to keep a defined study period and cohort population. Infections with finalized treatment prior to the inclusion period were not cause for exclusion.

The medical records of the identified 461 patients were manually reviewed at a minimum of five years after the primary revision procedure by the first author.

The primary revision procedure was defined as the first treatment procedure performed on the index prosthesis during the inclusion period, e.g. the procedure in which the infected implant was removed in a two-stage revision. The index prosthesis was defined as the hip joint replacement first treated for a chronic hip PJI during the inclusion period.

For each patient, data on comorbidity registered in a 5-year period prior to the primary revision procedure was obtained from the National Patient Registry for the estimation of the Charlson Comorbidity severity *(CCS)* score[12,13]. A re-infection after the primary revision procedure was defined according to Table 1.

Follow-up was done through the individual hospital patient-administrative-system and the nationwide electronic patient records. The electronic patient records were implemented nationally in 2009, and mandatorily register all outpatient and hospital visits based on the national civil registration system[14,15]. Thus, we were able to investigate if a patient had died and additional nationwide treatment performed on all included patients, with exact dates for these events. All medical records were available, except for one patient who emigrated (see Fig 1). Study approval was obtained from The Danish Health and Medicines Authority (3-3013-129/1/KAHO) and the Danish Data Protection agency (2010-41-4294). No formal, individual patient consent was required as study approval was obtained by the Danish Health and Medicines Authority (Health Act §42. 2). Nor was approval by an ethics committee required (The National Committee on Health Research Ethics, Committee act 2.7).

### Data analysis

Dichotomized data were compared by Chi-squared test and reported as proportions with a 95% confidence interval (CI), t-test for parametric data reported as means with a 95% CI, and rank-sum test for categorical or non-parametric data reported as medians with Interquartile Range (IQR). QQ-plots were assessed for normality. Re-infection was analyzed by competing risk analysis under the assumption of independent censoring[16]. Competing events were death and open aseptic revision. Competing-risk regression (Fine & Gray model) was fitted to examine predictor variables for re-infection. Proportional-Hazards assumption was assessed graphically.

Kaplan-Meier method was used to estimate cumulative all-cause mortality. A Cox regression model was fitted to examine predictor variables on mortality. Log-rank test was used to compare survival estimates.

Due to the potential relevance of the predictor variables, we choose to collapse age into 5-year intervals, Body Mass Index (BMI, kg/m^2^) into groups of *underweight* (BMI <18.5), *normal weight* (BMI 18.5–25), *overweight* (BMI 25–30), *severe overweight* (BMI >30) and CCS score into groups of *0 co-morbidity*, *1 co-morbidity* (equally ranked), *2 co-morbidities* (equally ranked) or *3+ co-morbidities* (equally ranked).

STATA 11.2 (STATA corp. College Station, TX) was used for all data analysis.

## Results

### Patient and surgical characteristics

Out of the 130 patients, 53 (41%) had a spacer in-situ, 64 (50%) had a resection arthroplasty and 13 (9%) did not have an exchange performed after the primary revision procedure (see Fig 1). The vast majority of spacers were constructed using Biomet^®^ hip spacer mold with Refobacin^®^ Revision or Palacos^®^ R-40 bone cement. All patients were operated via a posterior approach. A total of 82 (63%) of the 130 patients were eventually re-implanted in a two-stage revision. In these patients, re-implantation was performed after an interim period of 14 weeks (IQR 10–18).

*Staphylococcus sp*. was the pre-dominant microorganism grown in peri-operative tissue biopsies in the primary revision procedure (see Table 2). Thirty-two patients were culture-negative and 11 (32%) of these presented with a fistula. No differences between the groups regarding cultures were detected. All *Staph*. *aureus* were Methicillin sensitive. The antibiotic treatments were performed on a case-by-case decision based on available antibiogram and following the instructions from the local medical microbiologist. The majority of treatments involved Dicloxacillin and/or Cefuroxime as the main therapeutic drug. Duration of treatment was highly individual and based on a clinical response. The baseline differences between the established groups were significantly different regarding age, CCS score, BMI, HgB, and ASA score, but not in other registered variables (see Table 3).

**Table 2:** Microorganism cultured in 130 patients treated for chronic hip PJI between 2003 and 2008.

Microorganism cultured	Number (%)
Culture negative	32 (25)
*Staphylococcus aureus*	29 (22)
Coagulase-negative *Staphylococcus species*	26 (20)
*Streptococcus species*	12 (9)
*Enterococcus faecalis*	8 (6)
Miscellaneous species	8 (6)
*Proteus species*	5 (4)
Polymicrobial	5 (4)
*Pseudomonas aeruginosa*	2 (2)
No information available	3 (2)

**Table 3:** Baseline demographics of 130 patients treated for chronic hip Periprosthetic Joint Infection between 2003 and 2008.

Variable	Overall Cohort	Re-implanted	Not reimplanted	p-value
Age in years, mean (95%CI)	71 (69–73)	68 (66–71)	76 (72–80)	0.0006
Male gender, % (95%CI)	51 (42–59)	57 (46–68)	40 (26–55)	0.07
Excessive alcohol consumption*, % (95%CI)	10 (4–15)	12 (6–22)	4 (1–15)	0.16
Smoker, % (95%CI)	26 (19–34)	25 (15–35)	29 (15–42)	0.64
Antithrombotic treatment, % (95%CI)	30 (22–39)	32 (21–42)	29 (16–42)	0.76
SIRS at time of procedure˜, % (95%CI)	3 (0–6)	1 (0–4)	6 (1–13)	0.11
Index HJR is a revision prosthesis, % (95%CI)	25 (17–33)	25 (15–35)	24 (11–37)	0.86
Number of prior operations to index hip, median (IQR)	2 (1–3)	2 (1–3)	2 (1–4)	0.06
CCS, median (IQR)	0 (0–1)	0 (0–1)	1 (0–2)	0.005
In-situ duration of index prosthesis in weeks, median (IQR)	89 (37–241)	88 (38–229)	91 (27–317)	0.73
BMI groups, % (95%CI)				
<18.5 18.5–25 25–30 >30	4 (0–7) 46 (37–54) 29 (21–38) 21 (14–28)	4 (0–8) 33 (23–44) 40 (29–50) 23 (14–33)	5 (0–11) 68 (54–82) 11 (2–21) 16 (5–27)	0.001
Pre-operative hemoglobin in mmol/l, mean (95% CI)	7.3 (7.1–7.5)	7.6 (7.4–7.8)	6.8 (6.5–7.2)	0.0004
ASA score, median (IQR)	2 (2–2)	2 (2–2)	2 (2–3)	0.0001
Duration of surgery at primary revision procedure in minutes, mean (95%CI)	148 (137–159)	156 (141–170)	133 (115–151)	0.05
Blood loss at primary revision procedure in liters, mean (95%CI)	1.7 (1.5–1.9)	1.8 (1.6–2.1)	1.6 (1.3–2.0)	0.42
Number of blood transfusions following primary revision procedure, median (IQR)	4 (3–6)	4 (3–6)	4 (2–7)	0.75
Length of stay following primary revision procedure in days, median (IQR)	25 (18–41)	24 (18–39)	25 (19–46)	0.67
Follow-up in years, median (IQR)	8 (6–9)	7.9 (6.2–9.3)	8.7 (6.9–10.4)	0.03

SIRS: Systemic Inflammatory Response Syndrome; CI: confidence interval; IQR: Interquartile Range, Q1-Q3; ASA: American Society of Anesthesiologists score; BMI: Body Mass Index; CCS: Charlson Comorbidity severity score; HJR: Hip Joint Replacement;

* More than 21 units/week for men and 14 units/week for women.

˜ 2 or more of: temperature >38.0/<36.0, Heart rate >90/min, Respiratory rate >20/min, White blood cell count >12.0x10^9^/<4.0x10^9^

### Re-infection

**Fig 2:**
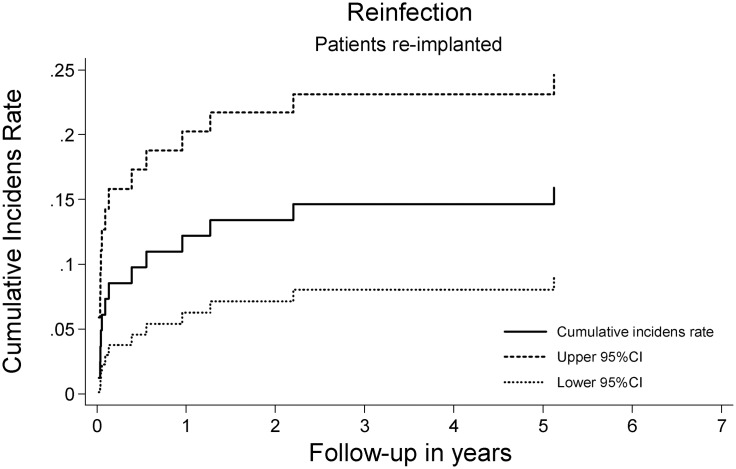
Re-infection. Cumulative incidence curve on re-infection after treatment for chronic periprosthetic hip joint infection in 81 patients undergoing re-implantation following a two-stage revision strategy in the presence of competing events, death and open aseptic revision.

The re-infection rate in the *re-implanted cohort* at five years was 14.6% (95%CI 8.0–23.1). There were no registered re-infections beyond six years of follow-up (Fig 2). Neither Gender, age, CCS, ASA, BMI or PJI category were identified as predictors of re-infection in the competing risk regression modeling.

### Mortality

The overall 1-year survival rate was 92% (95%CI 86–96) and the overall 5-year survival rate was 68% (95%CI 59–75). In the eighth follow-up year the survival rate declined below 50%.

**Fig 3:**
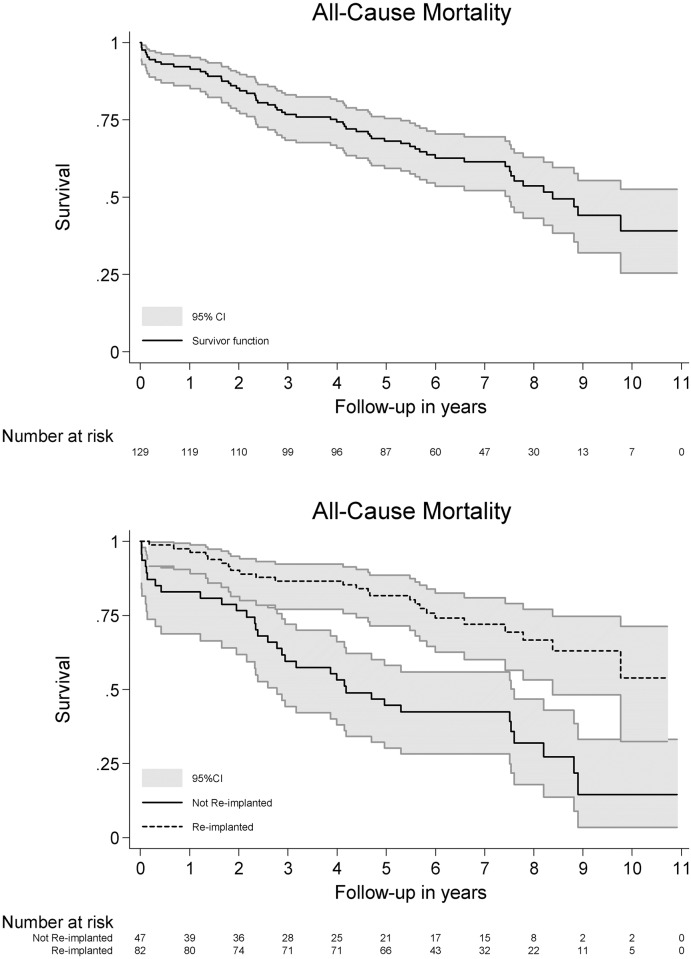
Mortality. Survival curve after treatment for chronic periprosthetic hip joint infection in 130 patients (upper). Survival curves after treatment for chronic periprosthetic hip joint infection in 81 patients undergoing re-implantation following a two-stage revision strategy and 48 patients not undergoing re-implantation following a two-stage revision strategy (lower).

The 1-year survival rate in the *non re-implanted cohort* was 83% (95%CI 69–91) and in the *re-implanted cohort* 98% (95%CI 91–99). The 5-year survival rate in the *non re-implanted cohort* was 45% (95%CI 30–58) and in the *re-implanted cohort* 82% (95%CI 71–89). Survival curves for all-cause mortality are shown in Fig 3.

There was a significant un-adjusted difference in survival between the two groups (hazard ratio 0.32: 95%CI 0.10–0.53; p-value <0.00001). After adjusting for gender, age group, ASA, CCS, underweight and pre-operative hemoglobin level, the difference was no longer significant (adjusted hazard ratio 0.75; 95%CI 0.30–1.87; p-value 0.54). Independent predictors of mortality during the follow-up period in the established cohorts are shown in Table 4.

**Table 4:** Cox regression model fitted on selected predictive variables for assessment of influence on survival regardless of treatment received in 130 patients treated for chronic hip PJI between 2003 and 2008.

Variable		Hazard Ratio	95% Confidence Interval	P-value
CCS*	Crude Adjusted	1.83 1.68	1.46–2.29 1.31–2.17	<0.0001 <0.0001
Female vs. Male	Crude Adjusted	1.27 0.97	0.76–2.13 0.53–1.77	0.37 0.93
Age*	Crude Adjusted	1.33 1.29	1.17–1.52 1.11–1.50	<0.0001 0.001
Normal BMI* vs. underweight	Crude Adjusted	2.30 13.97	0.81–6.55 3.44–56.71	0.12 0.002
Normal BMI vs. overweight	Crude Adjusted	0.68 0.70	0.48–0.97 0.46–1.06	0.03 0.09
HgB	Crude Adjusted	0.63 0.94	0.48–0.84 0.70–1.32	0.002 0.72
ASA	Crude Adjusted	3.63 2.69	2.26–5.84 1.50–4.82	<0.0001 0.001

HgB: pre-operative hemoglobin level; ASA: American Society of Anesthesiologists score; BMI: Body Mass Index CCS: Charlson Comorbidity severity score. Multi-variable analysis are adjusted for Gender, Age, ASA, CCS, Hgb

*Collapsed variable: age in 5-year intervals; BMI *underweight* (<18.5), *normal weight* (18.5–25), *overweight (*>25); CCS *0 co-morbidity*, *1 co-morbidity* (equally ranked), *2 co-morbidities* (equally ranked), *3+ co-morbidities* (equally ranked).

## Discussion

We report on a "non-selected" cohort of patients undergoing inpatient treatment for a chronic hip PJI, and examined the treatment strategy and prognosis of these patients.

We found that only 63% of patients had a re-implantation performed. Published reports describe re-implantation rates up to 92% or often do not state it[17–22]. The cause of the difference may pertain to the fact that our patient population is a non-selected sample, whereas in other studies, patients are referred to tertiary referral centers, reporting on their specialized experiences.

The flow of patients, who by all causes do not get re-implanted, could influence head-to-head comparisons especially between observational studies due to selection bias [3–5]. Patients re-implanted clearly differed from those not re-implanted by being younger, having a lower CCS, a higher BMI, a higher pre-operative hemoglobin level and lower ASA scores; indicating that patients undergoing re-implantation in a two-stage revision are a highly selected population. Based on the findings in this study, it is clear that the reporting on revision strategies in chronic hip PJI in current literature often lacks a sufficient description of treatment allocation to enable a proper comparison between treatment strategies[3].

We found that approximately 15% of patients in our two-stage revision group were re-infected within five years. In 2014, Zeller et al[22] reported on the prognosis following treatment for chronic hip PJI from a tertiary referral center using identical inference methods. The vigorous treatment protocol in this center, lead to an impressive five percent re-infection rate, which must set a benchmark in PJI treatment. We found female gender to be the only predictor of re-infection in our sample population. Other studies[21,23] have highlighted gender, presence of a fistula, inadequate antimicrobial treatment, and microorganism as potential predictors of re-infection, but these results could not be confirmed by our study. Most studies are restricted to predictors of hip PJI following primary procedures, and the investigation of the predictors for re-infection following treatment for chronic hip PJI is somewhat inhibited by the relatively few cases and large diversity in patient demographics. The vast majority of re-infections occurred within three years post-operatively, which appears as a relevant time frame for future studies to use as a minimal follow-up period.

We established an overall high mortality in our cohort. More than 50% of patients had died within eight years of follow-up. Others have commented on the potential correlation between patients with a hip PJI and mortality rates [17,18]. Mortality rates up to 48% at 5-year follow-up have been reported, and these are significantly different in comparison to aseptic revisions[18]. It is plausible that patients with a chronic hip PJI have increased risk of dying or vice versa. However, we cannot comment on the causality of hip PJI and mortality in our cohort as we do not have the cause of death, or have compared to a matched background population.

Mortality may bias results between treatment strategies. Berend et al[17] recently highlighted one aspect of this, and concluded that control of infection is not achieved if a patient is not re-implanted, and that future reports should include such a "worst-case" scenario.

Whether patients in our cohort were selected for a treatment strategy, due to co-morbidities or risk of dying at the time of decision, or that patients simply die before being offered a chance for re-implantation is beyond the limits of this study. But it is indicated, that patients re-implanted had a lower risk of dying compared to those not re-implanted. By inspection of the survival curve in Fig 3, it is clear that the *non re-implanted cohort* experience an early and rapid decline in the survival curve. We found higher ASA score, higher CCS score, higher age at time of primary revision procedure and underweight as predictors of mortality. Other studies have found divergent results. Choi et al[18] identified only CCS score as a predictor of mortality, whereas ASA score, age, gender were not predictive. CCS score was repeatedly identified by Zmistowski[24], but they also identified age as a predictor. Further investigation into these predictors is warranted on larger populations.

This study has some limitations. This is not a nested cohort, and misclassification is a concern. Patients not registered correctly may differ systematically from those correctly registered. Due to the sample size significant finding should be interpreted with caution due to the risk imposed by the issues of *multiple comparison testing*. By the retrospective nature of this study, information bias pertaining to information obtained in the medical records review may exist. This study is biased by *immortal person time* as we estimated time-at-risk from date of re-implantation. *Immortal person time* is the time from removal of index prosthesis to re-implantation. During this time period patients cannot die. This leaves a theoretical disadvantage concerning mortality incidence rates, as the *re-implanted cohort* would implicitly be older by the time frame of the interim period. We did perform sensitivity analysis with and without *immortal person time* and the estimated rate differences were interpreted to have no impact on the study conclusions.

A strength of this study is the use of competing risk analysis. Risk estimates represent a simple way of reporting data, however to optimize the use of all available patient data from longitudinal studies, time-to-event analysis should be performed. Only a limited number of studies on the prognosis following treatment for chronic hip PJI use this concept[20–22]. In future studies, performing inference by competing risk analysis could potentially lead to an increased quality of comparison between different treatment strategies and centres.

We believe this study confirms that selection exists when choosing patients for re-implantation, and that this must be taken into consideration when head-to-head comparisons on revision strategies are done based on observational studies. An elaborate description of the overall sample from which the study population was assembled, to enable a more precise comparison is preferable.

We found a high mortality rate in our sample population, but the causality of death and chronic hip PJI cannot be established in this current study and warrants further investigation, which we plan to perform in near future.

## Supporting Information

S1 File Stata datafile. Contains study data.
(DTA)
